# Use of propofol for procedural sedation reduces length of stay in the emergency department

**DOI:** 10.1186/cc12328

**Published:** 2013-03-19

**Authors:** J Millar, F Adamson, P O'Connor, R Wilson, E Ferrie, R McLaughlin

**Affiliations:** 1 Royal Victoria Hospital, Belfast, UK

## Introduction

Procedural sedation is used in the emergency department (ED) to facilitate short but painful interventions. Many patients are suitable for discharge after completion. Ideally, the agent used to achieve sedation should not have a prolonged effect, allowing safe discharge in the shortest time frame. We hypothesised that propofol, with its short onset and offset, may reduce length of stay (LOS) in comparison with traditional benzodiazepines.

## Methods

Data from a prospective registry were analysed for the period 1 August 2011 to 31 January 2012. Patients who underwent procedural sedation and who were discharged from the ED were identified. Individuals were grouped as having received propofol, midazolam or a combination of the two. All were discharged when fully alert and able to eat and drink. Demographic details and the type of procedure undertaken were extracted. ANOVA was performed to identify differences in the length of stay between groups, in addition to descriptive analysis.

## Results

**Figure 1:**
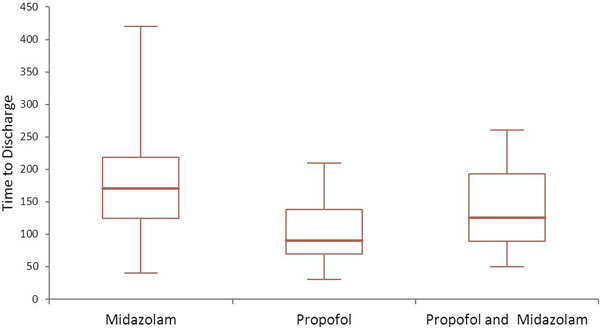
**Length of stay (minutes) in the ED**.

During the study period 75 patients underwent procedural sedation and were discharged from the ED. The median age was 40 years and 57% were male. The commonest procedure performed was shoulder reduction (52%). In the propofol group (*n = *20) the mean LOS was 100 minutes compared with 165 minutes in those receiving midazolam (*n = *40) and 141 minutes in those receiving a combination (*n = *15), *P *= 0.004. There was no difference in adverse events between groups. See Figure 1.

## Conclusion

Propofol is increasingly used in EDs for procedural sedation due to its short duration of action. This study suggests that a shorter duration of action and faster recovery may result in a reduced LOS in the ED.

